# Pan-NLRome of *Spinacia* facilitates the rapid discovery of downy mildew resistance genes

**DOI:** 10.3389/fpls.2026.1766206

**Published:** 2026-02-10

**Authors:** Hongbing She, Huiyu Wang, Zhaosheng Xu, Helong Zhang, Zhiyuan Liu

**Affiliations:** 1 State Key Laboratory of Vegetable Biobreeding, Institute of Vegetables and Flowers, Chinese Academy of Agricultural Sciences, Beijing, China; 2 Zhongyuan Research Center, Chinese Academy of Agricultural Sciences, Xinxiang, China; 3 Beijing Key Laboratory of New Technique in Agricultural Application, Beijing University of Agriculture, Beijing, China

**Keywords:** downy mildew, *k*-mer-based GWAS, pan-NLRome, RPF1, spinach

## Abstract

Plant disease resistance is typically conferred by nucleotide-binding site leucine-rich repeats (NLR) proteins; however, the diversity of NLR genes in spinach has remained largely unexplored. We identified 2,549 NLR genes across 19 *Spinacia* assemblies of cultivated spinach and its two wild species, and constructed a comprehensive pan-NLRome, which was categorized into six subfamilies, and the most frequent NLR class was CC-NBARC-LRR. The pan-NLRome consists of 186 NLR families, comprising 38.7% core, 51.1% dispensable and 10.2% private families. By integrating pan-NLRome with *k*-mer-based genome-wide association studies (GWAS), we developed a novel pipeline for rapid identification of disease resistance genes. Using this approach, we directly pinpointed a candidate gene, *Te17S24XX_Chr1_nlr42*, for the *RPF1* locus, which confers resistance to spinach downy mildew pathogen races 1–7, 9, 11, 13, 15, 16, 18, and 20. In contrast, a single-genome-based method identified four candidate genes, which required further analysis confirm the final gene. The *Spinacia* pan-NLRome serves as an invaluable resource for exploring NLR gene evolution and plant disease resistance mechanisms. Our developed pipeline offers a reliable and efficient strategy for cloning resistance genes across multiple crops.

## Introduction

Plants have evolved a two-tiered immune system to defend against diverse pathogenic invasions. The first layer, known as pattern-triggered immunity (PTI), is activated when pattern recognition receptors (PRRs) localized on the cell surface detect pathogen-associated molecular patterns (PAMPs). The second layer of immunity, termed effector-triggered immunity (ETI), is mediated by intracellular nucleotide-binding site leucine-rich repeats (NLRs) (Dangl et al., 2013; Wang et al., 2022). NLRs recognize specific pathogen effectors, thereby leading to a hypersensitive response (HR) to restrict pathogen growth (Van de Weyer et al., 2019). The N-terminal domain is usually a Toll/interleukin-1 receptor/resistance protein (TIR) or a coiled-coil (CC) (Ameline-Torregrosa et al., 2008; Shao et al., 2016). Numerous studies have demonstrated the crucial role of NLRs in plant resistance, as evidenced in *Brachypodium* (Wu et al., 2022), wheat (Li et al., 2024; Ning et al., 2025), and rice (Wang et al., 2015).

Genome-wide association studies (GWAS) have become a faster approach to identifying statistical associations between single-nucleotide polymorphisms (SNPs) and phenotypic traits across diverse populations (Shang et al., 2023; Liu et al., 2024). However, this approach is limited by its inability to capture the full spectrum of genetic variation, particularly presence-absence variants (PAVs) and copy number variations (CNVs) (Zhou et al., 2022; Meng et al., 2025). This limitation is particularly pronounced in disease resistance research, as resistance (*R*) genes are frequently located in genomic regions rich in structural variation (Van de Weyer et al., 2019). To overcome the limitations, *k*-mer-based GWAS has emerged as a powerful reference-free approach for trait mapping (Voichek and Weigel, 2020). This method directly processes sequencing reads, comparing the diversity of *k-*mers within the population, thereby avoiding alignment biases introduced by reference genomes. The *k*-mer-based GWAS have proven highly effective in disease resistance, often directly linking loci with phenotypes that standard SNP-based GWAS fail to detect, such as in maize, tomato (Voichek and Weigel, 2020), and wheat (Arora et al., 2019; Jaegle et al., 2025). Furthermore, compared to a single reference, the pan-genomes more fully represent the entire genetic information of a species (Zhou et al., 2022). Therefore, by utilizing a pan-genome as the foundation for *k*-mer analysis, more novel loci can be captured. For instance, based on the pan-genomes, 93% of powdery mildew resistance-associated *k*-mers were identified in wheat, uncovering more than 25% *k*-mers compared to methods using a single reference genome (Jaegle et al., 2025). Recently, pan-NLRome has been constructed in numerous plant species (Mo et al., 2024; Ning et al., 2024, 2025; Parada-Rojas et al., 2025), laying the foundation for investigating disease resistance mechanisms.

Spinach downy mildew, caused by *Peronospora effusa* (Pe), formerly known as *Peronospora farinosa* f. sp. *spinaciae* (*Pfs*), is one of the most destructive diseases of spinach worldwide (Qian et al., 2016; Ribera et al., 2020). A total of 20 races were reported since 1824, 16 of which have increased substantially from 1996 to 2024 (Feng et al., 2014, 2018b; Correll et al., 2024). Six spinach downy resistance genes/alleles (*RPF1*–*RPF6*, resistance against *Peronospora Farinose*) have been reported, which could be overcome by specific races of the pathogen (Correll et al., 2011). For example, the *RPF1* locus provides resistance to races 1–7, 9, 11, 13, 15, 16, 18, and 20, while *RPF3* resists races 1, 3, 5, 8, 9, 11, 12, 14, 16, and 19 (Bhattarai et al., 2023). Previous studies have demonstrated that the *RPF1*–*RPF3* locus is located in the 0.34–1.76 Mb on chromosome 3 of the Sp75 genome assembly (Feng et al., 2018a; She et al., 2018; Bhattarai et al., 2020; Gao et al., 2022; Bhattarai et al., 2023). Specifically, the *RPF2* was reported to be the 1.11–1.72 Mb on chromosome 3 of Sp75 assembly, a CC-NBS-LRR domain gene *Spo12821* serving as the potential candidate gene for *RPF2* (Gao et al., 2022; Bhattarai et al., 2023). We previously fine-mapped the *RPF1* locus to 0.89 Mb of Sp75 chromosome 3 between 0.34–1.23 Mb using BC_1_ and F_2_ population (She et al., 2018), consistent with the 0.84 Mb interval detected using genotyping by sequencing (GBS) based SNP markers (Bhattarai et al., 2020). Although two candidate NLR genes, *Spo12903* and *Spo12784*, for *RPF1* were obtained (She et al., 2018), the key gene has yet to be determined.

Here, we identified comprehensive NLRs in 19 representative spinach assemblies and constructed a pan-NLRome. Based on the pan-NLRome, we optimized the *k*-mer-based GWAS approach to develop a pipeline for rapidly identifying disease-resistant genes/loci. Using this approach, we identified the candidate gene *Te17S24XX_Chr1_nlr42* (formerly termed *Spo12903* in Sp75 assembly) for the downy mildew resistance gene *RPF1* in spinach. Together, our work provides a foundation for identifying and functionally investigating disease resistance genes.

## Materials and methods

### NLR identification and classification

To obtain comprehensive NLR from spinach, we identified NLR across 19 representative *Spinacia* assemblies using NLR-Annotator v. 2.1b (Steuernagel et al., 2020) with default parameters. The NLRs in spinach could be further divided into six subfamilies: CC-NBARC-LRR, CC-NBARC, NBARC-LRR, NBARC, TIR-NBARC, TIR-NBARC-LRR. To assess the distribution of NLRs, we visualized them using the telomere-to-telomere genome (Sp_YY_v2) as an example.

Based on the result from NLR-Annotator, we also obtained each NLR and its flanking sequences (2 kb) for each genome, generating pan-NLRome sequences.

### Gene prediction

To further determine NLRs and their corresponding protein-coding genes, we performed automatic structural gene annotations for 19 *Spinacia* assemblies using Helixer v0.3.6 (Holst et al., 2023), as previous studies have demonstrated its exceptional accuracy in annotating NLR genes (Belinchon-Moreno et al., 2025). We employed an in-house script to identify NLR and its overlapped protein-coding genes, which were then subjected to further analysis.

### Gene-based pan-NLRome construction

We constructed a pan-NLRome using the NLR protein-coding genes from 19 *Spinacia* assemblies. First, we clustered these genes using OrthoFinder (Emms and Kelly, 2015) with the default parameters, and then divided these gene families into three categories, core, dispensable, and private based on their frequency. We defined the NLR families as core, dispensable, and private if they were present in all 19 accessions, 3–18 accessions, and 1–2 accessions, respectively.

### *K_A_*/*K_S_* analysis of pan-NLRome

For comparison of the *K_A_*/*K_S_* value between the core and dispensable NLR families, we first selected the longest length NLR protein-coding gene from each accession within both the core and dispensable NLR families as the representative gene. Subsequently, TBtools (Chen et al., 2020) was employed to calculate the *K_A_*/*K_S_* value for homologous gene pairs between each pair of genomes within both the core and dispensable NLR families.

### *K*-mer-based GWAS

We utilized 116 spinach accessions (She et al., 2024a), comprising 30 resistant and 86 susceptible to *Peronospora effuse* race 9 (*Pfs9*), to identify causal variants associated with resistance to *Pfs9* based on our optimized *k*-mer-based GWAS. First, we filtered low-quality short reads from 116 accessions using fastp v0.23.4 (Chen et al., 2018) with the parameter “-q 20”. Then, the clean reads were aligned to pan-NLRome sequences using BWA 0.7.17-r1188 (Li, 2013) with default parameters. After removing duplicated reads, we obtained the paired-end (PE) reads that were located at pan-NLRome sequences (termed NLR-reads) using SAMtools (Li et al., 2009) with the parameters “view –F 12 –q 30”.

We created the *k*-mers table as described in Jaegle et al. (2025). Specifically, we firstly extracted *k-*mers (31 bp) from NLR-reads for each accession using KMC (Kokot et al., 2017). Then, we combined and filtered lists of *k*-mers using the script list_kmers_found_in_multiple_samples with parameters “--mac 5 –p 0.2”. Last, we obtained the *k*-mers table, containing the presence/absence pattern of each *k*-mers in the 116 spinach accessions. Combining the phenotype and *k*-mer table, we run *k*-mer-based GWAS with a permutation-based threshold for 5% family-wise error rate.

To retrieve the coordinates of the significant *k*-mers, we first extracted the PE reads containing the significant *k*-mers using fetch_reads_with_kmers (https://github.com/voichek/fetch_reads_with_kmers). Subsequently, we quantified the number of PE reads containing significant *k*-mers on pan-NLRome sequences to identify the target NLR or determined the coordinates of the significant *k*-mers by aligning the corresponding PE reads against the target genome using BWA 0.7.17-r1188 (Li, 2013) with default parameters.

### RNA-seq analysis

We aligned the clean reads against the Sp_YY_v2 genome using HISAT2 v2.1.0 (Kim et al., 2015), followed by calculating the read count using featureCounts v2.0.1 with the parameters ‘-T 10 -p -t exon -g ID’ (Yang et al., 2014). Gene expression was normalized using the transcripts per million (TPM) method with an in-house script. The expression patterns are shown using R v4.1.1.

### Genome-wide association studies

The 116 spinach accessions mentioned above were used for SNP-based GWAS analysis based on the Sp_YY_v2 assembly. The clean reads were aligned to Sp_YY_v2 assembly using BWA 0.7.17-r1188 (Li, 2013) with default parameters. The SNPs were identified using GATK v4.3 (McKenna et al., 2010), and then filtered using the GATK with “QD < 2.0 | | FS > 60.0 | | MQ < 40.0| | SOR > 3.0 | | MQRankSum < −12.5 | | ReadPosRankSum < −8.0”, and indels with “QD < 2.0 | | FS > 200.0 | | SOR > 10.0 | | MQRankSum < −12.5 | | ReadPosRankSum < −8.0; (2)”, and VCFtools v0.1.16 (Danecek et al., 2011) with the parameters “--max-missing 0.85 --mac 4 --minQ 30 --maf 0.05 --min-alleles 2 --max-alleles 2”. A total of 1,087,947 high-quality SNPs were obtained. The GWAS was performed using EMMAX software (Kang et al., 2010).

## Results

### Identification of nucleotide-binding site and leucine-rich repeat genes in 19 *Spinacia* genomes

**Figure 1:**
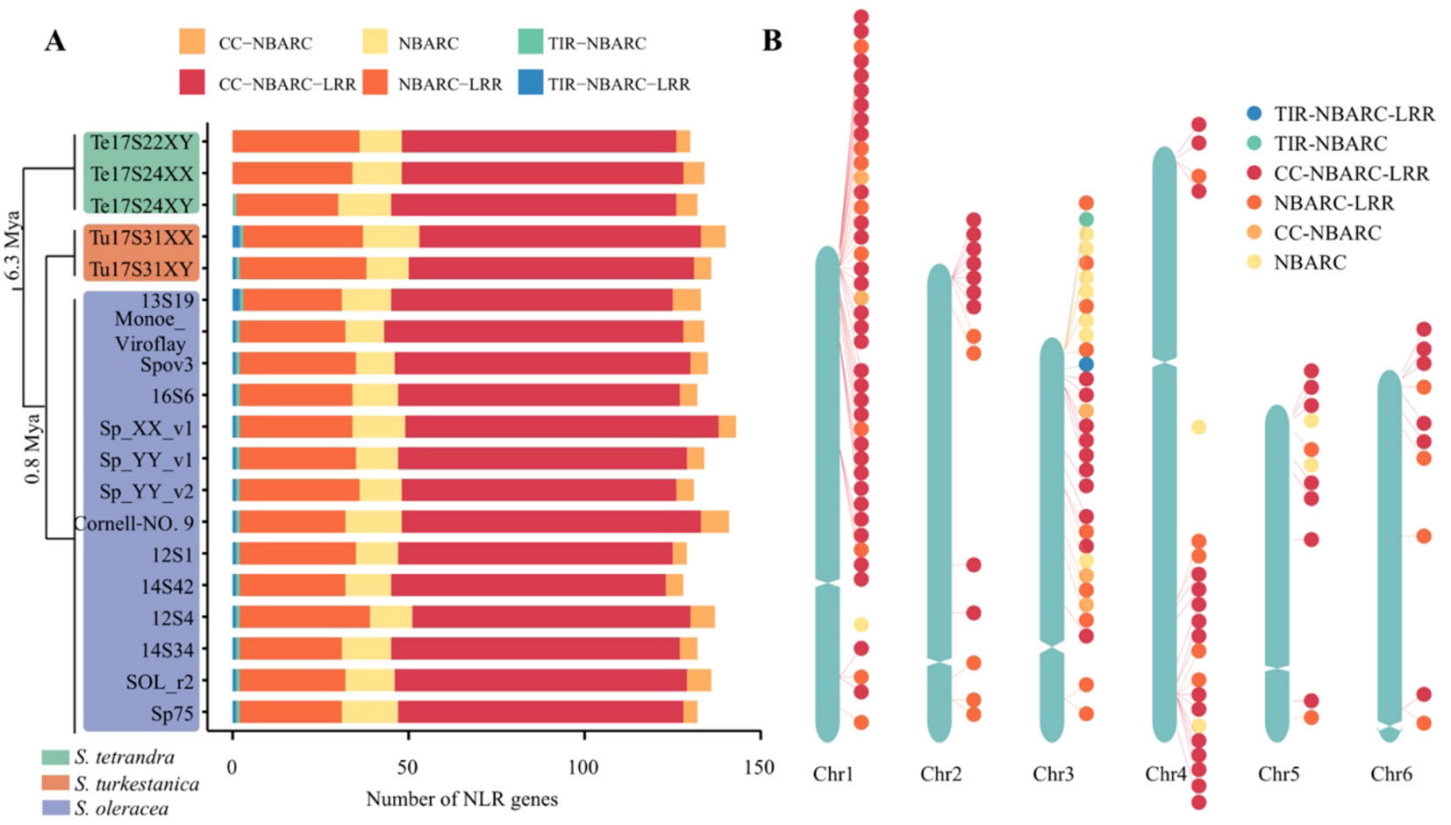
Overview of NLR genes in the 19 *Spinacia* accessions. **(A)** Number of six subfamilies of NLR genes in the 19 *Spinacia* assemblies. The divergence times for the three *Spinacia* species were inferred in our previous study (She et al., 2024b). **(B)** Distribution of NLR genes in Sp_YY_v2 telomere-to-telomere (T2T) genome.

To determine the dynamics in different *Spinacia* genomes, we predicted NLR genes in 19 *Spinacia* genomes using NLR-Annotator (Steuernagel et al., 2020). Fourteen spinach genomes were from our previous studies (She et al., 2023, 2024a, 2024b); the remaining five assemblies (Monoe-Viroflay, SpoV3, Cornell-No.9, SOL_r2, and Sp75) were used in others (Xu et al., 2017; Cai et al., 2021; Hirakawa et al., 2021; Hulse-Kemp et al., 2021; Ma et al., 2022). These accessions included 14 cultivated spinach species, *S. oleracea*, two of its closest wild relatives, *S. turkestanica* (0.8 million years ago (Mya)), and three more distant relatives, *S. tetrandra* (~6.3 Mya) (**Figure 1A**; **Supplementary Table S1**).

In total, 2,549 NLR genes were annotated in the 19 spinach genomes, with a range of 128 to 143 genes per accession (**Supplementary Table S2**). The 12 cultivated spinach plants and their five wild relatives shared a similar number of NLR genes (Wilcoxon test, *p* = 0.89) (**Supplementary Figure S1**). Furthermore, these NLR genes were categorized into six subfamilies, and the most frequent NLR class was CC-NBARC-LRR (60.58%), followed by NBARC-LRR (23.90%) and NBARC (9.96%) (**Figure 1A**, **Supplementary Table S2**). TIR-NBARC and TIR-NBARC-LRR are rarely detected in *Spinacia* species, particularly in *S. tetrandra* species (**Supplementary Table S2**), suggesting that the two subfamilies probably evolved after splitting from *S. tetrandra* and *S. turkestanica*/*S. oleracea*. NLRs exhibited an uneven distribution along chromosomes, invariably clustering together and predominantly located near subtelomere regions (**Figure 1B**). Applying the definition of NLR clusters as genes within 200 kb of each other in the genome (Holub, 2001), 47.73%–57.86% of NLRs in each accession were located in such clusters (**Supplementary Table S3**, **Supplementary Figure S2**), consistent with a previous report on *Arabidopsis thaliana* (Van de Weyer et al., 2019).

### Pan-NLRome of *Spinacia*

**Figure 2:**
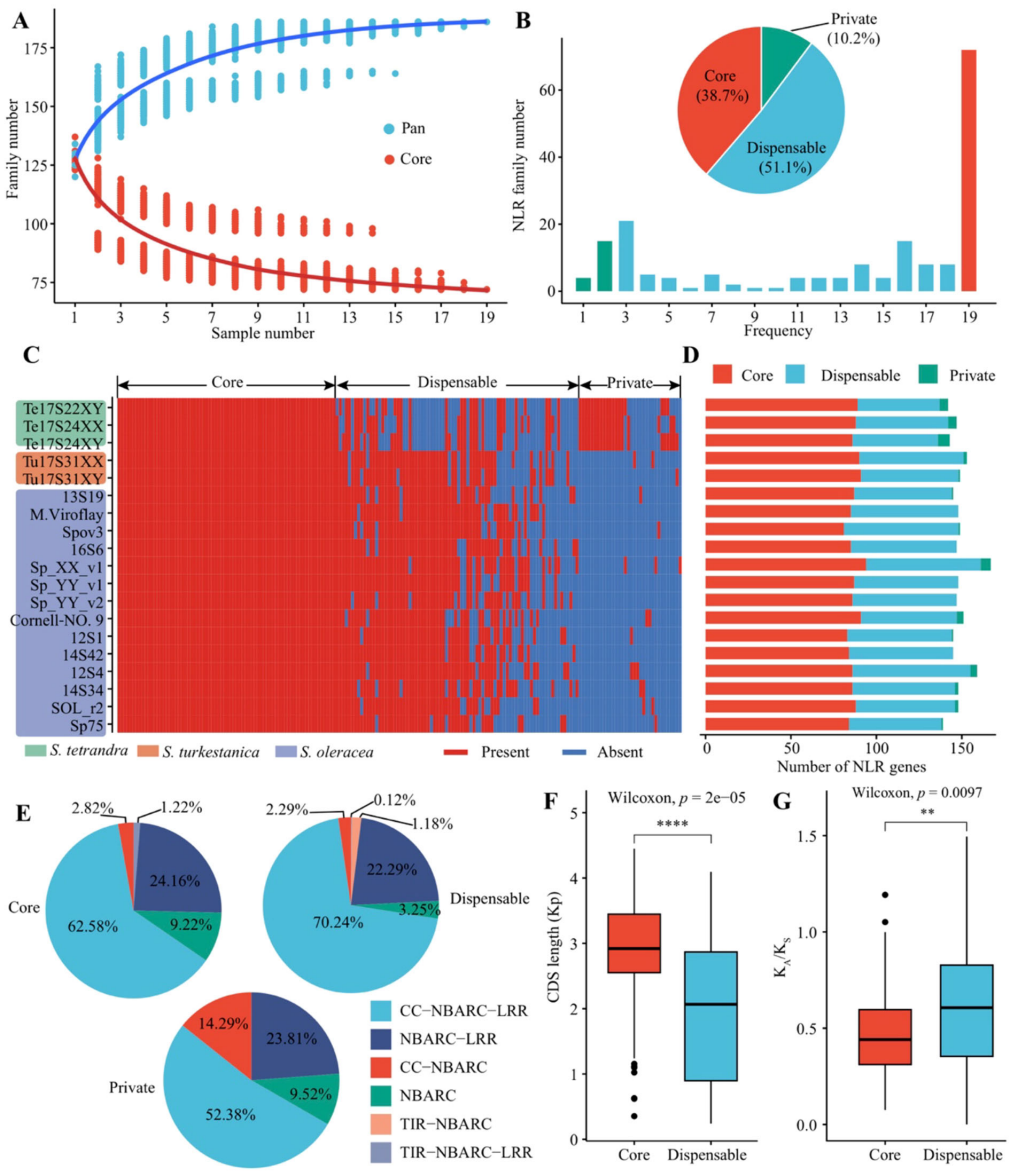
Pan-nucleotide-binding site leucine-rich repeat (NLR) analysis. **(A)** Pan- and core NLR of 19 *Spinacia* accessions. The blue and red curves represent the number of pan- and core NLR families after random combinations for each specific number of accessions. **(B)** Distribution of NLR families in the pan-NLRome. The histogram shows the number of NLR gene families in the 19 genomes with different frequencies. The pie chart shows the proportion of the NLR gene family in the core (red), dispensable (blue), and private (green) NLR genes. **(C)** Presence and absence information of pan NLR gene families in the 19 *Spinacia* genomes. **(D)** The number of core, dispensable, and private NLR genes of each genome. **(G)** Proportion of different NLR gene types in core, dispensable, and private NLR genes, respectively. Comparison of CDS length **(E)** and *K_A_*/*K_S_***(F)** between the core and dispensable NLR genes, respectively. Significance was determined using the Kruskal-Wallis test. **p < 0.01, ****p ≤ 0.0001. For each boxplot, the box edges represent the interquartile range (IQR), with the centerline indicating the median. The whiskers extend to 1.5× the interquartile range.

To understand the variation in NLR content, we inferred 2846 NLR protein-coding genes that overlapped with NLR loci in the corresponding assembly. 2813 (99%) of these genes were annotated and associated with disease resistance using the Non-redundant protein Sequence (NR), Swiss-Prot, Pfam, TrEMBL, and *A. thaliana* databases (**Supplementary Table S4**). Then, we identified 186 pan-NLRome by clustering NLR protein-coding genes (see Methods) in the 19 *Spinacia* accessions (**Supplementary Table S5**). The number of NLR families exhibits a positive correlation with genome number, with their abundance stabilizing at a genome number of 12 (**Figure 2A**), indicating that the 19 *Spinacia* accessions are diverged and that the pan-NLRome closely captures the NLRs of spinach. Furthermore, only one and none additional NLR family was found when the 12^th^ and 17^th^ accession was added, respectively (**Supplementary Figure S3**).

Based on the frequency of NLR gene families in the 19 *Spinacia* genomes, we further divided the pan-NLRome into core, dispensable, and private families. In total, we obtained 72 (38.7%) core NLR families present in 19 accessions, 95 (51.1%) dispensable NLR families present in 3–18 accessions, and 19 (10.2%) NLR families present in 1–2 accessions (**Figure 2B**). The proportion of pan-NLRome gene families across 19 genomes revealed that dispensable NLRs are more likely to be present in *S. oleracea*/*S. turkestanica* compared to *S. tetrandra*, whereas private NLRs showed the opposite trend (**Figure 2C**), suggesting high diversity between *S. oleracea*/*S. turkestanica* and *S. tetrandra*. Moreover, the 19 spinach accessions shared an average of 58.99% of core NLR genes, exceeding the proportion found in dispensable NLR genes (39.93%) (**Figure 2D**, **Supplementary Table S6**). These dispensable NLR genes were significantly prevalent in *S. oleracea*/*S. turkestanica* (Wilcoxon test, *p* < 0.01); conversely, the private NLR genes (1.48%) were predominantly present in the *S. tetrandra* (Wilcoxon test, *p* < 0.05) (**Supplementary Figure S4**), further confirming that *S. tetrandra* represents a more distant spinach wild relative.

Among the core, dispensable, and private NLR genes, the most frequent NLR class was CC-NBARC-LRR (52.38%–70.24%), followed by the NBARC-LRR (22.29%–24.16%) and NBARC (3.25–9.52%) (**Figure 2E**). The proportion of CC-NBARC in private NLR genes (14.29%) was higher than that in core (2.82%) and dispensable (2.29%) NLR genes (**Figure 2E**; **Supplementary Table S7**). Moreover, the core NLR genes exhibited longer CDS length (Wilcoxon test, *p* < 2e-5) and lower *K_A_*/*K_S_* values (Wilcoxon test, *p* < 0.0097) compared to dispensable NLR genes, suggesting that core NLR genes possess more conserved functions, consistent with previous findings in soybean (Liu et al., 2020) and broomcorn millet (Chen et al., 2023).

### A pipeline for identifying resistance genes using pan-NLRome and *k*-mer-based genome-wide association studies

**Figure 3:**
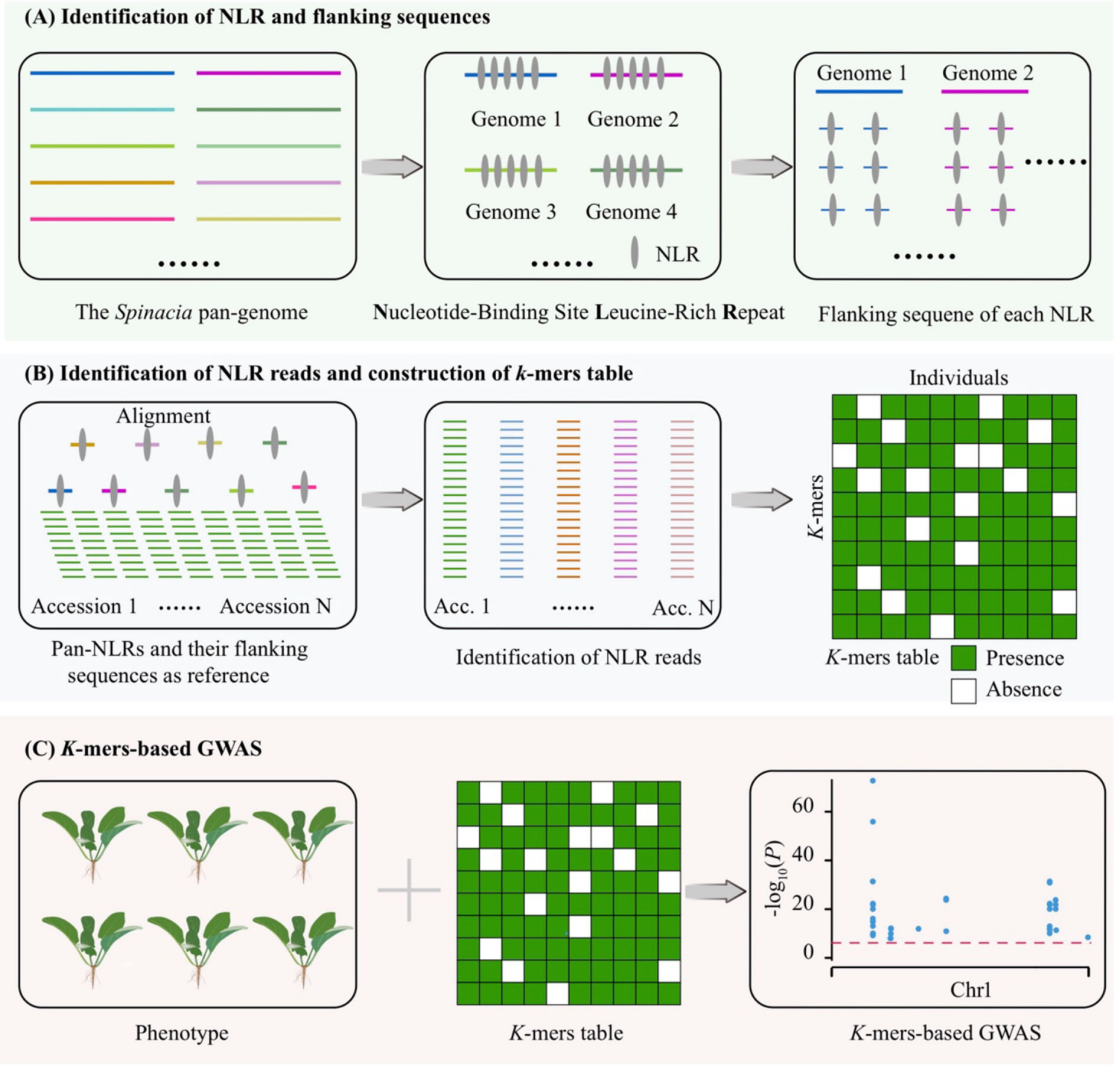
Workflow for identifying resistance genes using *k*-mer-based GWAS. **(A)** Identification of nucleotide-binding site leucine-rich repeat (NLR) in 19 *Spinacia* assemblies. **(B)** Identification of NLR reads and *k*-mers table. We first aligned paired-end (PE) reads to each NLR and its flanking sequences (2 kb) and obtained mapped PE reads. Then, we extracted *k*-mers (31 bp) of each accession and constructed a *k*-mers table. **(C)** We performed GWAS based on phenotype and *k*-mers table as described by Voichek and Weigel, 2020.

*K*-mer-based GWAS is an efficient approach to identify causal variants associated with phenotypes, particularly disease resistance in plants (Voichek and Weigel, 2020; Jaegle et al., 2025). To enhance the efficiency of disease-resistance gene discovery, we optimized this approach by integrating the pan-NLRome, which primarily consists of three steps: identification of NLR and flanking sequences (**Figure 3A**), identification of NLR reads and construction of *k-*mer table (**Figure 3B**), and conduction of *k-*mer-based GWAS (**Figure 3C**). First, we obtained the nucleotide-binding site leucine-rich repeat (NLR) and its flanking sequences (2 kb) for each of the 19 *Spinacia* genomes, generating a pan-NLRome sequences. Secondly, we identified NLR reads by aligning short reads of spinach accessions to pan-NLRome sequences, followed by extracting *k*-mers (31 bp) and constructing a *k*-mers table. Last, we performed *k*-mer-based GWAS based on phenotype and *k*-mer table as described by Voichek and Weigel, 2020.

### Screening of downy mildew resistance gene *RPF1* in spinach

**Figure 4:**
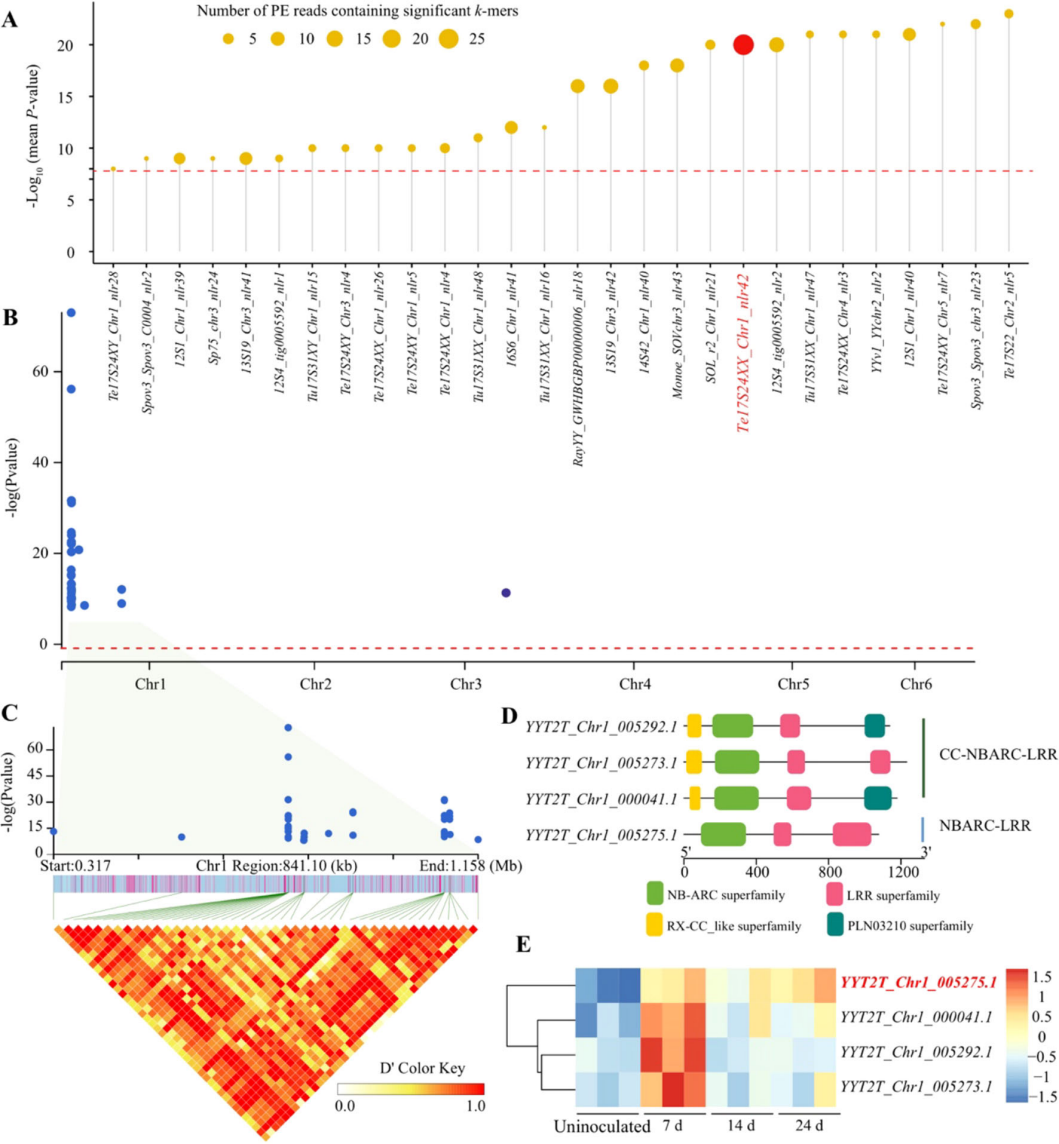
Identification of the downy mildew resistance gene *RPF1* in spinach. **(A)** The–Log_10_(mean *P*-value) of *k*-mers on pan-NLRome sequences. Dot size represents the number of PE reads containing significant *k*-mers. The red horizontal dashed line indicates the Bonferroni-corrected significance thresholds (a = 0.05). **(B)** Manhattan plot of *k*-mer-based GWAS of downy mildew resistance gene *RPF1*. Only significantly *k*-mers were shown. **(C)** The expanded version of the significant *k*-mers and LD pattern in Chr1. **(D)** The conserved domains of four candidate genes of *RPF1*. **(E)** Expression patterns of the four candidate genes of *RPF1* in uninoculated plants and those inoculated with downy mildew pathogen *Pfs9* at 7, 14, and 24 days post-inoculation. The gene labeled in red indicates that its expression level was significantly higher than that in uninoculated plants across all three post-inoculation periods.

To demonstrate the usefulness of our approach, we utilized 116 spinach accessions (**Supplementary Table S8**) (She et al., 2024a), comprising 30 resistant and 86 susceptible to *Peronospora effuse* race 9 (*Pfs9*), to identify downy mildew resistance gene *RPF1* loci that resist *Pfs9* (Correll et al., 2011; She et al., 2018). Among these accessions, a total of 111.02 Gb of short reads were obtained from the NLR and flanking sequences, generating a *k*-mer table comprising 10,001 *k*-mers. In our *k*-mer-based GWAS, we detected 50 significant *k*-mers associated with *RPF1*, with the *Te17S24XX_Chr1_nlr42* gene enriching the highest number of *k*-mers and exhibiting high significance. (**Figure 4A**). Notably, our previous study identified *Te17S24XX_Chr1_nlr42* (formerly designated as *Spo12903*) as a candidate gene for *RPF1* using BC_1_ and F_2_ populations (She et al., 2018).

Otherwise, to assess the effectiveness of *k*-mer-based GWAS in a single genome, we mapped the PE reads containing the significant *k-*mers against the Sp_YY_v2 assembly. The vast majority of these reads were located within the 781 kb–1101 kb interval on chromosome 1 (**Figures 4B, C**, **Supplementary Table S9**), consistent with previous observations (She et al., 2018; Bhattarai et al., 2020; Cai et al., 2021). Within the candidate region, we identified four NLR genes, three of which carry the canonical CC-NBARC-LRR architecture, and one gene, *YYT2T_Chr1_005275.1*, a homolog of *Te17S24XX_Chr1_nlr42* mentioned above, lacks the N-terminal coiled-coil domain (NBARC-LRR only) (**Figure 4D**). The expression analysis indicates that all four genes exhibited elevated expression levels at 7 days post-inoculation, with only the *YYT2T_Chr1_005275.1* gene maintaining sustained expression at both 14 and 24 days (**Figure 4E**). Therefore, we confirmed that *Spo12903* is probably *RPF1*.

Furthermore, we performed the SNP-based GWAS using the Sp_YY_v2 reference genome, which successfully detected significant SNPs encompassing the *YYT2T_Chr1_005275.1* locus (**Supplementary Table S10**). However, the SNP-GWAS also identified numerous other significant signals across the genome, making it difficult to unequivocally prioritize this specific gene (**Supplementary Figure S5**). Taken together, compared to a single genome, pan-NLRome enables faster and more accurate anchoring of candidate genes.

## Discussion

Nucleotide-binding site leucine-rich repeat (NLR) proteins represent one of the most important gene families in plants, as they confer disease resistance by recognizing pathogen proteins (Van de Weyer et al., 2019). Although 139 NBS-LRR genes have been reported previously in Sp75 assembly (Xu et al., 2017), this is far from sufficient. A single genome cannot capture the entirety of spinach genetics, particularly lacking genetic sequences from wild species, which are considered donors of downy mildew resistance loci for cultivated spinach (She et al., 2024b). In this study, we identified 2,549 NLR genes in the 19 spinach genomes, including 14 cultivated spinach, two of its closest wild relatives, *S. turkestanica*, and three more distant relatives, *S. tetrandra* (**Figure 1A**, **Supplementary Table S2**). Furthermore, we constructed a pan-NLRome including 186 NLR gene families in *Spinacia*, which were classified into six categories, consistent with the previous findings based on a single genome (Xu et al., 2017), but fewer subfamilies than in grape, which identified two additional subfamilies: TIR-CC-NBAARC-LRR and TIR (Guo et al., 2025).

*K*-mer-based GWAS serves as a powerful tool for identifying disease-resistance-associated genes and has been widely applied in various plants, such as tomato, maize (Voichek and Weigel, 2020), and wheat (Jaegle et al., 2025). Generally speaking, pan-genome captures missing heritability better than a single genome (Zhou et al., 2022). A recent study confirmed that pan-genome-based *k*-mer GWAS approaches can identify 25% more *k*-mers associated with powdery mildew resistance than single-reference methods (Jaegle et al., 2025). Using this reasoning, we developed a pipeline for rapidly identifying resistance-associated genes based on pan-NLRome and *k*-mer-based GWAS (**Figure 3**). This is because we extracted *k*-mers solely from paired-end (PE) reads within the NLR regions, utilized pan-NLRome to retrieve significantly associated *k-*mers, and ultimately directly obtained associated NLR genes. pan-NLRome *k*-mer-based GWAS rapidly identifies a candidate gene (*Te17S24XX_Chr1_nlr42*) for *RPF1*, while a single-genome-based approach detects four candidate genes, which require expression or functional analysis to determine the final gene, although both methods ultimately identify the same gene (**Figure 4**). Notably, the candidate gene *Te17S24XX_Chr1_nlr42* was identified in the wild relative, *S. tetrandra*, indicating that the downy mildew resistance loci in cultivated spinach were introgressed from wild species, corroborating our earlier conclusions (She et al., 2024b).

As the number of sequenced genomes increases, we foresee that our approach will become highly prevalent for the rapid identification of disease resistance genes, as it directly targets the gene of interest rather than the linked region. However, our pipeline is specifically designed for NLR disease resistance genes; otherwise, candidate genes would be overlooked. For other gene types, we strongly recommend utilizing the pan-genome to retrieve the location of significant *k*-mers, as described by Jaegle et al. (2025). Overall, our study offers a novel approach for rapidly identifying disease-resistance genes, laying the foundation for further elucidating their mechanisms.

## Conclusions

We constructed the comprehensive pan-NLRome for the genus *Spinacia*, revealing an extensive diversity of 2,549 NLR genes. The pan-NLRome provides a crucial framework for understanding the evolution and architecture of the immune system in spinach and its wild relatives. Then, we developed a novel pipeline that integrates the pan-NLRome with *k*-mer-based GWAS. This strategy allows for the rapid and precise identification of resistance genes, overcoming the limitations of traditional single-genome methods that produce ambiguous candidate lists requiring lengthy subsequent validation. The power of this approach is demonstrated by the direct identification of *Te17S24XX_Chr1_nlr42* as the candidate for the downy mildew resistance locus *RPF1* in spinach. Our findings provide valuable resources for exploring NLR gene evolution and plant disease resistance mechanisms, while offering a reliable and efficient strategy for cloning resistance genes in a wide range of crops.

## Data Availability

The datasets presented in this study can be found in online repositories. The names of the repository/repositories and accession number(s) can be found in the article/**Supplementary Material**.
