# Supplementary material for: Pan-NLRome of Spinacia facilitates the rapid discovery of downy mildew resistance genes
Source: Front Plant Sci. 2026 Feb 10;17:1766206. doi: 10.3389/fpls.2026.1766206 (PMC12929410; doi:10.3389/fpls.2026.1766206)
Supplement: Supplementary file 2 [file Table2.docx]

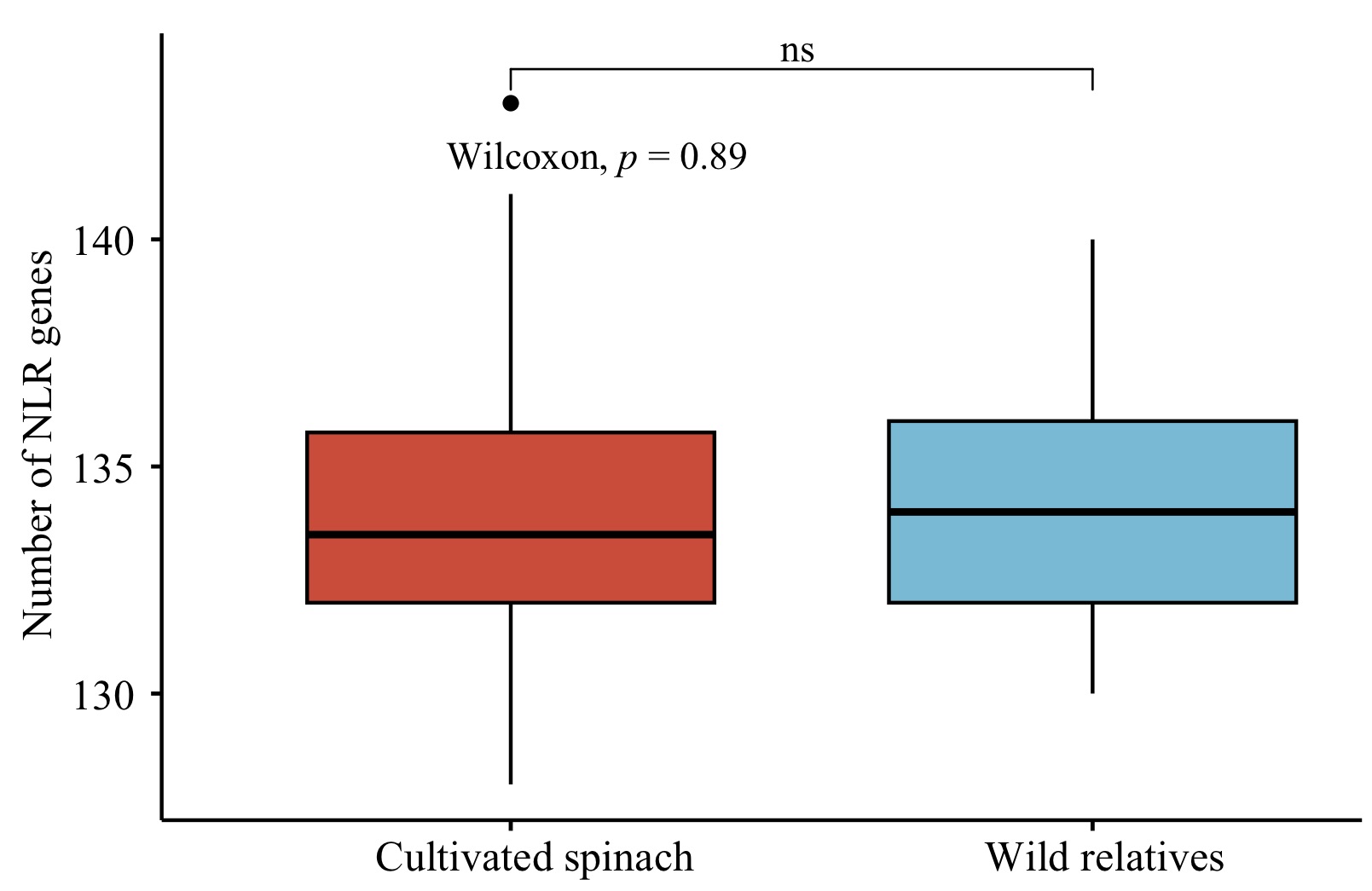


**Supplementary Figure S1.** Comparison of the number of NLR genes in cultivated spinach and its wild relatives. The lower and upper edges represent 25% and 75% quartiles, respectively, and central lines indicate the median. The whiskers extend to 1.5× the interquartile range. Significance was determined using the Wilcoxon test. ns, not significant.


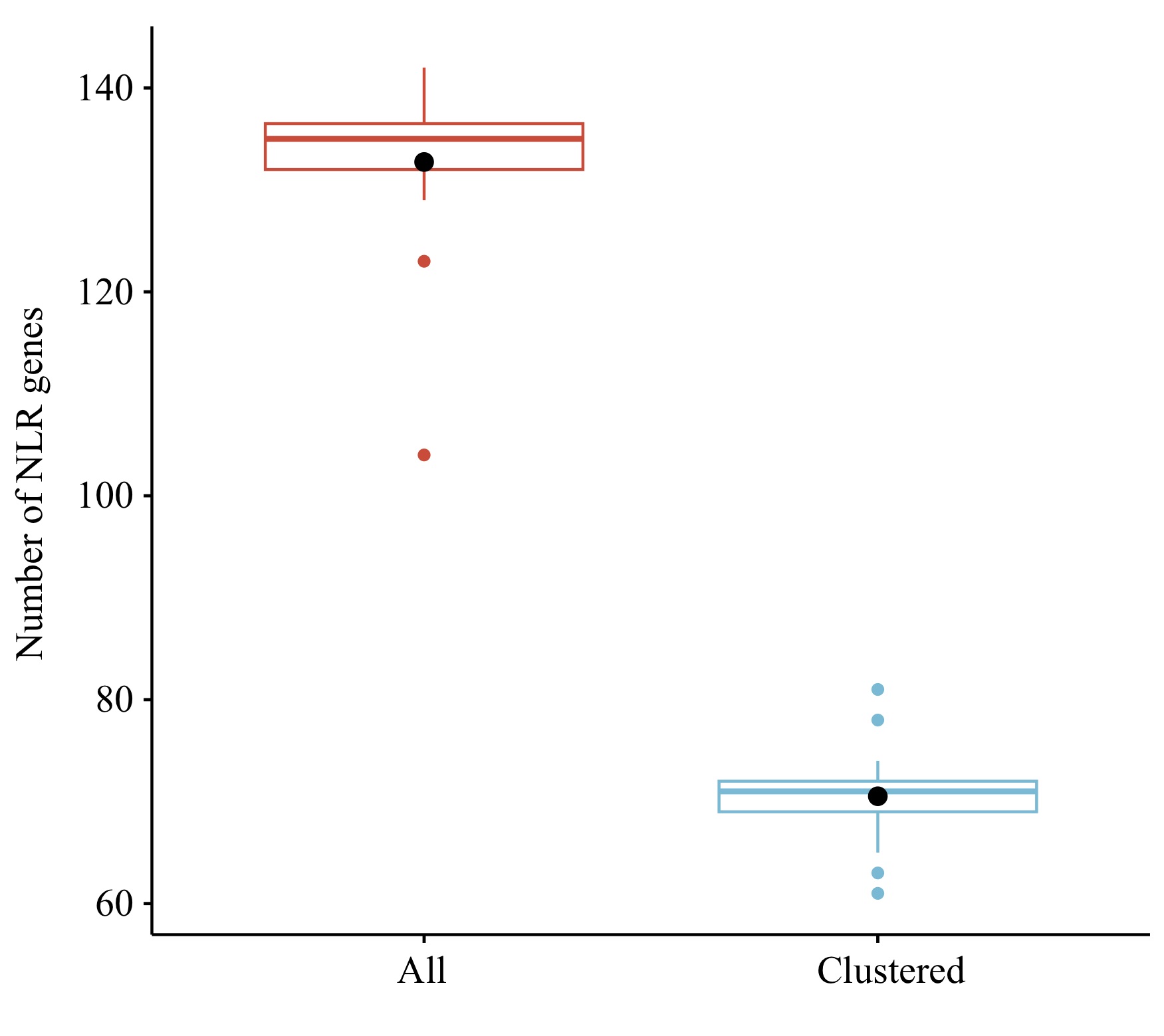


**Supplementary Figure S2.** Total number as well as number of clustered NRs in each accession. The lower and upper edges represent 25% and 75% quartiles, respectively, and central lines indicate the median. The whiskers extend to 1.5× the interquartile range. The black dots indicate the mean value.


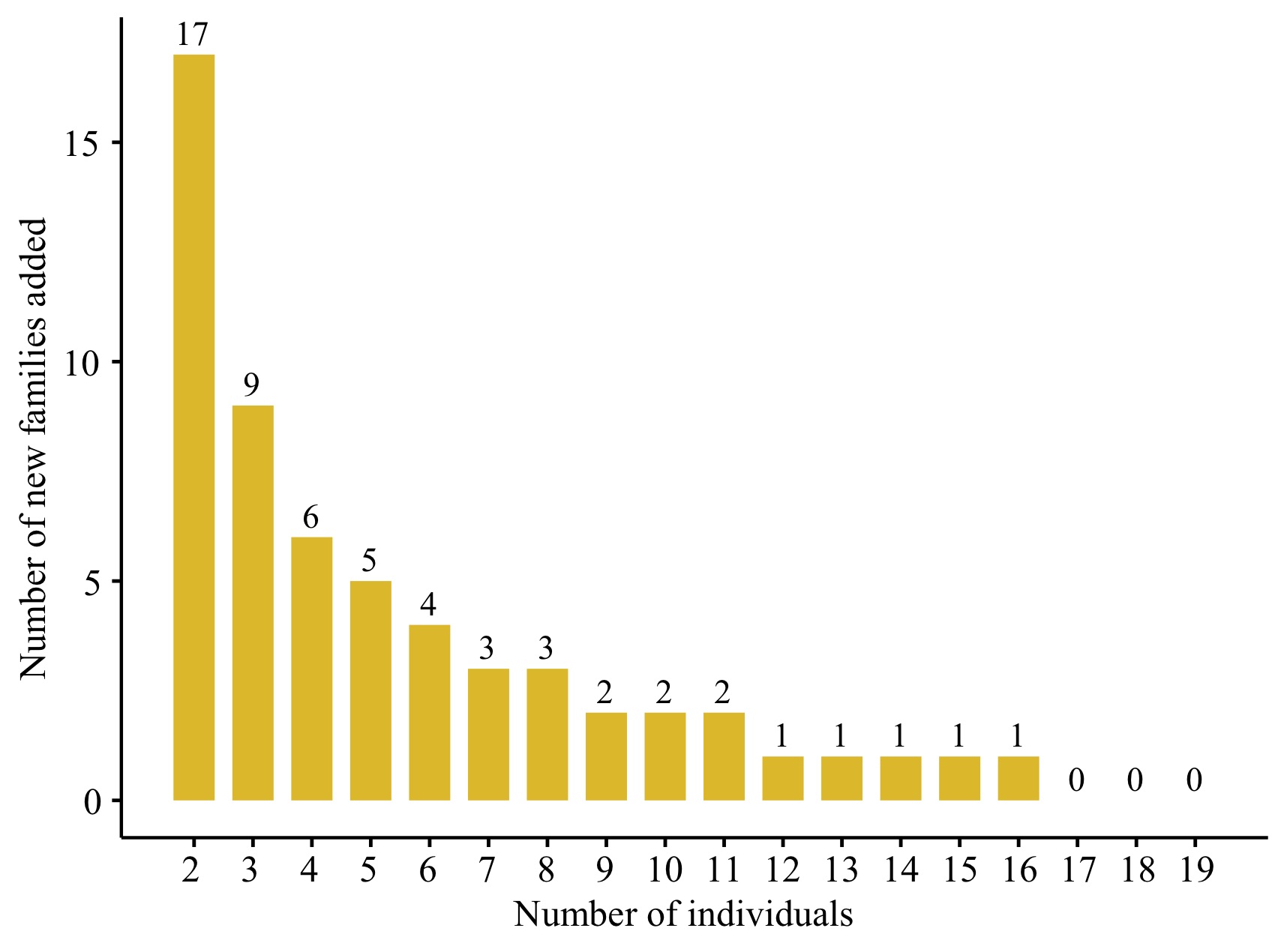


**Supplementary Figure S3.** Number of newly added NLR gene families when adding more spinach individuals.


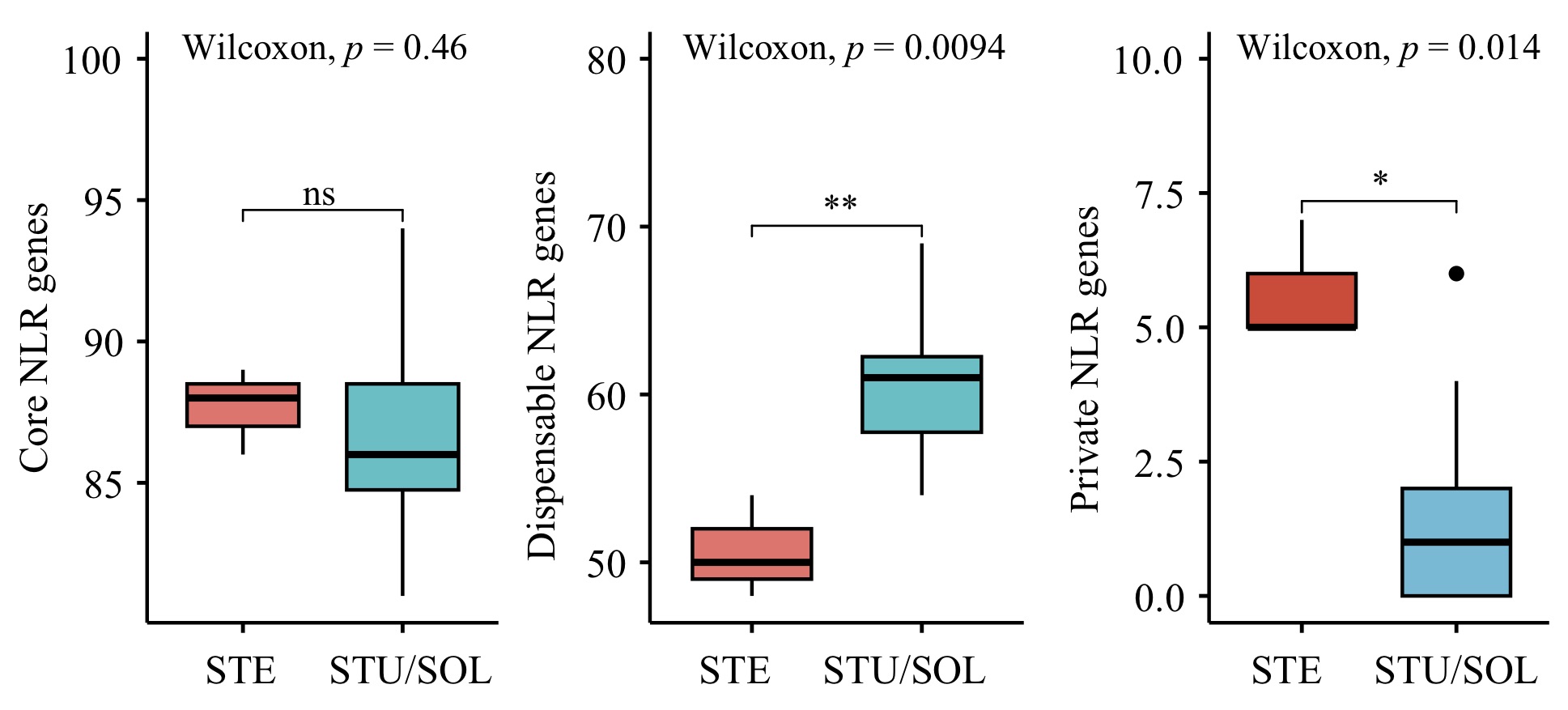


**Supplementary Figure S4.** Comparison of the number of pan-NLR genes among the three *Spinacia* species. STE, *S. tetrandra*, STU, *S. turkestanica*, SOL, *S. oleracea*. Significance was determined using the Wilcoxon test. ns, not significant; **p* < 0.05; ***p* < 0.01. The lower and upper edges represent 25% and 75% quartiles, respectively, and central lines indicate the median. The whiskers extend to 1.5× the interquartile range.


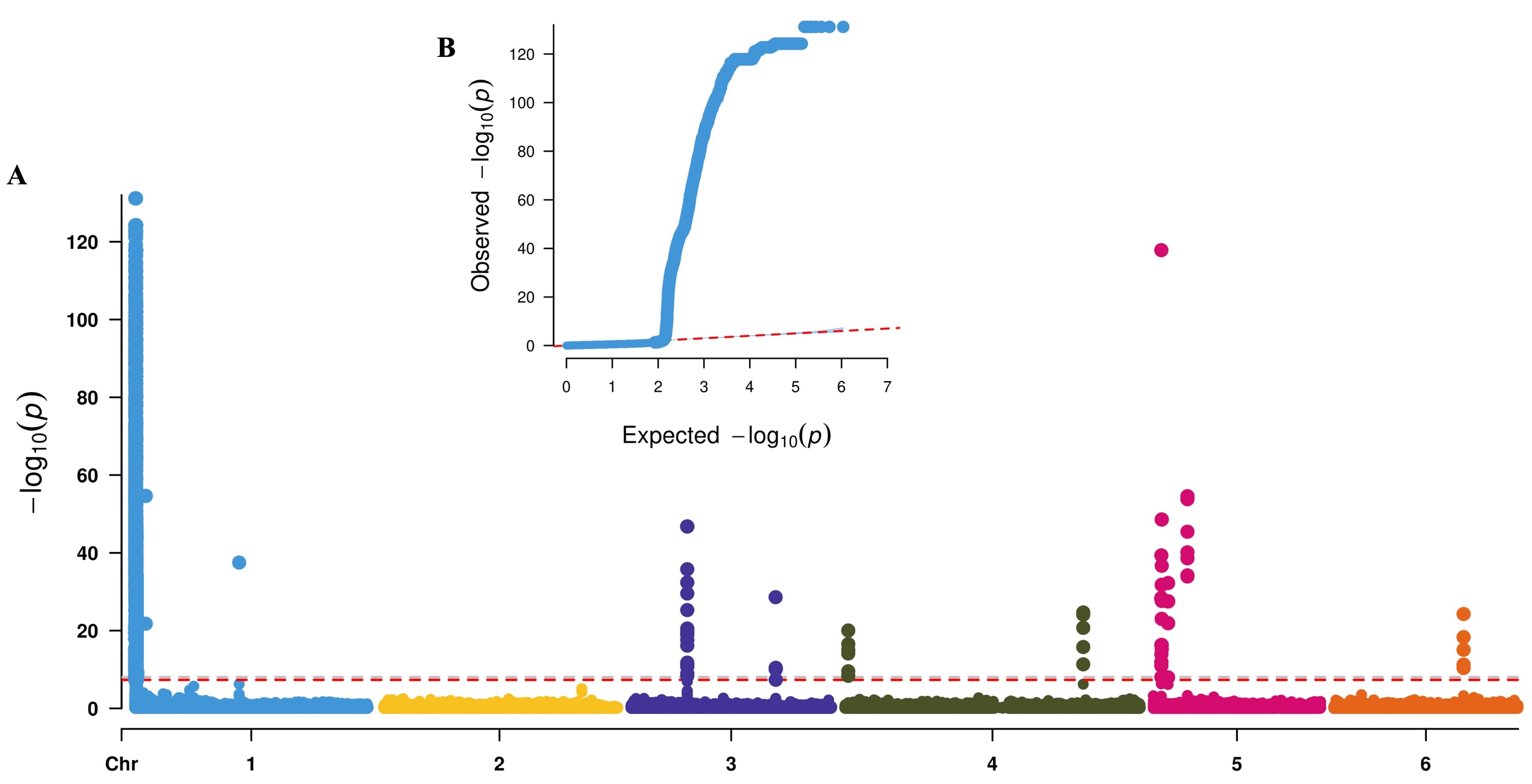


**Supplementary Figure S5.** GWAS results of the *RPF1* based on the SNPs dataset. Manhattan **(A)** and QQ plot **(B)** of GWAS *RPF1* based on the SNPs on the Sp_YY_v2 assembly. The grey and red horizontal dashed lines represent the Bonferroni-corrected significant thresholds of the GWAS (a = 0.01 and a = 0.05, respectively).
